# A Survey Report on Effect of Root Canal Fillings and Coronal Restorations on the Periapical Status of Endodontically Treated Teeth in a Selected Group of Population

**DOI:** 10.5005/jp-journals-10005-1196

**Published:** 2013-08-26

**Authors:** Poorva Khullar, Deepak Raisingani, Shailendra Gupta, Rohit Kumar Khatri

**Affiliations:** 2nd Year Student (MDS), Department of Conservative Dentistry and Endodontics, Mahatma Gandhi Dental College and Hospital, Jaipur Rajasthan, India, e-mail: poorvakhullar13@gmail.com; Professor, Department of Conservative Dentistry and Endodontics Mahatma Gandhi Dental College and Hospital, Jaipur, Rajasthan, India; Professor and Head, Department of Conservative Dentistry and Endodontics, Mahatma Gandhi Dental College and Hospital, Jaipur Rajasthan, India; Assistant Professor, Department of Conservative Dentistry and Endodontics, Mahatma Gandhi Dental College and Hospital, Jaipur Rajasthan, India

**Keywords:** Apical periodontitis, Endodontic treatment, Coronal restoration, Root fillings

## Abstract

**Aim:** A cross-sectional radiographic orthopantomogram (OPG) survey was done to determine the prevalence of apical periodontitis (AP) in root canal–treated teeth in a selected population, and to evaluate the influence of the coronal restorations (CR) and root canal fillings on the periapical status.

**Materials and methods:** A total of 250 OPGs were evaluated out of which root canal treatment (RCT) had been performed in 438 teeth which was taken as the sample size.

**Statistical analysis used:** Chi-square test, odds ratio and p-values were recorded.

**Results:** The results showed that 53.1% of the endodontically treated teeth presented with AP radiographically. Incidence of AP among teeth with acceptable RCT (32.3%) was significantly lower than those suffering from unacceptable RCT (92.7%). Moreover, adequate CR demonstrated a significantly better periapical status (83.2%) compared to teeth with inadequate CR (37.2%). Also, the combination of adequate CR and adequate root filling resulted in significantly reduced incidence of AP (21.6%) compared to the presence of AP (97%) when both parameters scored as inadequate.

**Conclusion:** Results hence demonstrated that a well-performed RCT and well-sealing CR are both essential for the overall success of endodontic treatment. Therefore, a considerable improvement in the quality of RCT and CR in general dental practice is required to promote oral/periapical health.

**How to cite this article:** Khullar P, Raisingani D, Gupta S, Khatri RK. A Survey Report on Effect of Root Canal Fillings and Coronal Restorations on the Periapical Status of Endodontically Treated Teeth in a Selected Group of Population. Int J Clin Pediatr Dent 2013;6(2):89-94.

## INTRODUCTION

Success in endodontic treatment was originally based on the triad of debridement, thorough disinfection, and root canal filling with all aspects equally important. At present, successful endodontic treatment is based on broader principles. These include diagnosis and treatment planning; knowledge of anatomy and morphology; the traditional concepts of debridement, thorough disinfection and root canal filling, and the coronal restoration (CR).^1^

Coronal leakage has also been demonstrated to contribute to treatment failure. Maintaining an effective coronal seal and placing an appropriate CR should be considered an essential component of successful endodontic treatment. One study reported that good postendodontic restorations resulted in significantly more successful cases when compared with good endodontics (80 *vs* 75.7%) and poor restorations resulted in significantly more periradicular inflammation cases when compared with poor endodontics (30.2 *vs* 48.6%).^2^

Radiological examination is a main tool for a thorough exploration in dentistry.^3^ The radiographic quality of the endodontic treatment was significantly more important than the technical quality of the CR when the periapical status of endodontically treated teeth was evaluated.

Outcomes studies can be designed using two major approaches: Case-controlled or epidemiologic study. The outcome of RCT in case-controlled studies has yielded success rates up to 98%.^4^ The high rates of success reported in such studies is from a relatively small number of endodontic treatment cases and controls which are usually carried out by endodontic specialists or undergraduate students under strict operating conditions in a university clinic. Therefore, such studies may not represent the reality of treatment carried out in the general practitioners' clinic. Epidemiologic surveys assess a large number of RCTs performed by both general practitioners and endodontists; therefore they will yield success rates that more realistically represent the treatment outcomes in the general population.^5^Unfortunately, a high percentage of inappropriate RCTs chiefly performed by general practitioners have been reported in many surveys; i.e. 24.5 to 65.8% of the endodontically treated teeth presented with AP.^6-9^

The aim of this study was to investigate the technical quality of root fillings and CRs in root-filled teeth, their association with periapical status and prevalence of apical periodontitis (AP).

## MATERIALS AND METHODS

A total of 250 orthopantomograms (OPGs) were evaluated out of which root canal treatment (RCT) had been performed in 438 teeth which were taken as sample size for the study.

Endodontic treatments should have been done within the last 10 years.

### Radiographic Variables and Diagnostic Categories (Parameters Registrations and Codes)

***Apical Periodontitis***

(Ørstavik et al 1986)

1 = Absence (normal periapical structures or small changes in bone structure).

2 = Presence (changes in bone structure with some mineral loss, AP with well-defined radiolucent area or extensive/ severe periodontitis with exacerbating features).

Size of AP*

1= <3 mm

2= >3 mm and <5 mm

3= >5 mm

***Length of Root Filling–***

(DeMoor et al 2000)

1 = Adequate (<2 mm from, or flush with, the radiographic apex).

2 = Inadequate (>2 mm from the radiographic apex or overextended).

***Density of Root Filling–***

(Dugas et al 2003)

1 = Adequate (uniform density and adaptation of the filling to the root canal walls).

2 = Inadequate (visible canal space laterally along the filling, voids within the filling mass, or identifiable untreated canal).

***Coronal Restorations***

(Siqueira et al 2005)

1 = Adequate (radiographically intact restoration with no signs of leakage).

2 = Inadequate (radiographic signs of overhangs, open margins/recurrent decay, presence of temporary CR or no CR).

Rsults would be based on the following criteria:

- Patient age
- Sex
- Number of remaining teeth
- Endodontically treated teeth
- Teeth with AP
- Most commonly root canal-treated tooth
- Least commonly treated tooth
- Size of the periapical lesion.

Chi-square test and odds ratio were used for statistical analysis along with the p-value.

* If a multirooted tooth presented with different periapical status at different roots, the root canal with the most severe periapical condition was categorized.

† In cases of multirooted teeth, not all root canal fillings of such teeth were assessed separately but only the canal with the worst technical obturation quality.

## RESULTS

These 250 OPGs (438 teeth) were analyzed retrospectively and following results were obtained. The average patient age was 39 ± 11 years in case of males and 37 ± 11 years in case of females. Also, the number of males undergoing endodontic treatment was found to be more than the females. During the study, incidentally it was found that the average number of remaining natural teeth were similar in males and females which was 27. The number of missing teeth per person increased significantly with age. Also, RCT was found to be more common in maxillary teeth (52.2%) as compared to the mandibular teeth (47.7%). Tables 1 and 2 show the distribution of endodontically treated teeth by tooth type.

As seen from Tables 1 and 2, the number of endodontically treated teeth in maxilla is more than that in mandible. Also, maxillary as well as the mandibular molars are the most commonly root canal-treated teeth. Mandibular molars have the highest incidence of RCT followed by maxillary molars and maxillary incisors and mandibular canines are the least commonly treated teeth.

As seen from Tables 3 and 4, mandibular molars are the most prevalent teeth with AP followed by maxillary molars and maxillary incisors. Again, canines are the least affected teeth with AP.

**Table 1:** **Table 1:** Distribution of endodontically treated teeth (maxilla)

*Tooth type*		*Number*		*Percentage*
Incisors		69		30.1
Canines		20		8.7
Premolars		60		26.2
Molars		80		34.9
Total		229		100

**Table 2:** **Table 2:** Distribution of endodontically treated teeth (mandible)

*Tooth type*		*Number*		*Percentage*
Incisors		40		19.1
Canines		10		4.7
Premolars		56		26.7
Molars		103		49.2
Total		209		100

**Table 3:** **Table 3:** Distribution of endodontically treated teeth with apical periodontitis (maxilla)

*Tooth type*		*Number*		*Percentage*
Incisors		39		33.3
Canines		9		7.6
Premolars		27		23.07
Molars		42		35.8
Total		117		100

**Table 4:** **Table 4:** Distribution of endodontically treated teeth with apical periodontitis (mandible)

*Tooth type*		*Number*		*Percentage*
Incisors		18		15.5
Canines		7		6.03
Premolars		28		24.1
Molars		63		54.3
Total		116		100

Out of 438 endodontically treated teeth, inadequate length of root canal filling was found in 24.8% teeth against 75.1% teeth which had adequate length, inadequate density was found in 29.6% teeth in contrast to 70.3% teeth which had adequate density and inadequate CR was found in 31.2% teeth against 68.8% teeth which had adequate CR. The RCT was found to be complete in 68.4% and incomplete in 31.6% cases. AP was present in 53.1% cases and absent in approximately 46.9% of all the root canal-treated teeth. Also approximately 88% of these periapical lesions were smaller than 3 mm, 8.9% were in between 3 and 5 mm and 3.3% were more than 5 mm.

As seen from Table. 5 which evaluates various parameters of quality of RCT, out of the total number of endodontically treated teeth (438), AP was found in 53.1% teeth. A clear correlation was found between the prevalence of AP and length/density of root filling of the endodontically treated teeth (p < 0.001). Teeth that had root fillings with adequate length/density (acceptable RCT) were tested against any other combination of unacceptable RCTs. Both length and density were found adequate in 68.4% teeth; interestingly, 32.3% of these teeth had AP, significantly less than any other combination of parameters (p < 0.001). In the cases of unacceptable RCT, AP was present in 93.4% of the teeth. These findings confirm that adequate length and density of the root canal fillings significantly affected the periapical status (Table 5).

**Table 5:** **Table 5:** Quality of RCT and apical periodontitis

*Parameter*		*Total number of teeth*		*Total number of teeth (%)*		*Teeth with apial periodontitis*		*Teeth with apial periodontitis (%)*		*X_2_(df)*		*p-value*
Endodontically treated teeth (n = 438)		438		100		226		53.1		40.9 (3)		0.000
Adequate length/adequate density (acceptable RCT)		300		68.4		97		32.3				
Adequate length/inadequate density (unacceptable RCT)		23		5.2		19		82.6				
Inadequate length/adequate density (unacceptable RCT)		27		6.2		24		88.8				
Inadequate length/inadequate density (unacceptable RCT)		88		20.09		86		92.7				
Unacceptable RCT		138		31.6		129		93.4				

**Table 6:** **Table 6:** RCT quality and coronal restoration in relation to apical periodontitis

*Parameter*		*Total number of teeth*		*Total number of teeth (%)*		*Teeth with apical periodontitis*		*Teeth with apical periodontitis (%)*		*X_2_(df)*		*p-value*
Acceptable RCT/adequate coronal restoration		231		52.7		49		21.6		61.0 (3)		0.000
Acceptable RCT/inadequate coronal restoration		69		15.7		48		69.5				
Unacceptable RCT/adequate coronal restoration		70		15.9		63		90				
Unacceptable RCT/inadequate coronal restoration		68		15.5		66		97				

It was also found that approximately 37.2% cases of adequate CR had AP against 83% cases of inadequate CR which had AP. The parameters for the combined quality of the CR and RCT are also shown in Table. 6. Both these variables were adequate in only 231 teeth (52.7%) and approximately one-fourth of these teeth (21.6%) had AP. When tested against other combinations of the quality of parameters, the acceptable CR and RCT combined category was significantly better than the others (p < 0.001). Conversely, when the CR and RCT were unacceptable (68 teeth), 97% of the endodontically treated teeth had AP (p < 0.001). Hence, it was confirmed that the quality of RCT and the quality of CR had a significant influence on AP (Table 5). The odds of finding AP in unacceptable RCT/ inappropriate CR cases was >5 times greater than acceptable RCT/appropriate CR.

**Fig. 1:**
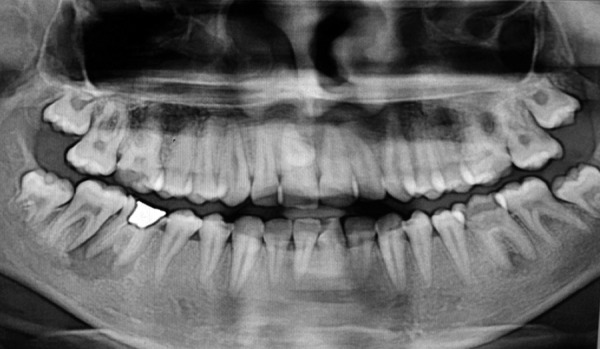
The effect or coronal restoration and root canal filling can be further evaluated with the OPGs shown here. Due to inadequate root canal fillings and improper CR, a periapical lesion more than 3 mm is seen in relation to mandibular right molar

**Fig. 2:**
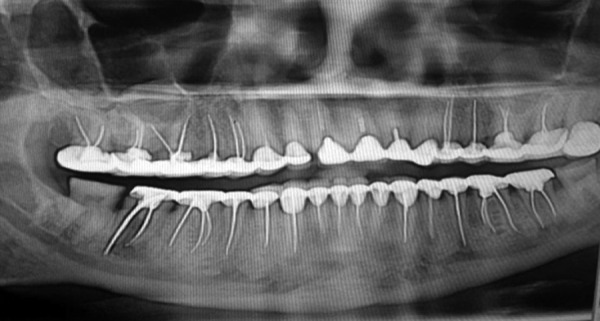
As seen from the above OPG, the root canal treated teeth have been adequately filled and restored with crowns. Hence, chances of finding AP in such cases automatically reduce to almost zero

Figures 1 and 2 show the examples of the OPGs used for the study and how the effect of root canal filling and CRs affect the prognosis of the tooth.

## DISCUSSION

Endodontic epidemiological studies conducted with different populations worldwide have shown that the periapical health status is related to factors like quality of root canal filling, CR and presence of an intracanal post.^6,10-13^ Therefore, root canal filling represents an important phase of endodontic treatment, which is completed with the placement of an adequate CR.

Using a cross-sectional model, this study evaluated the prevalence and risk factors of AP in endodontically treated teeth in a selected group of population on the basis of radiographic survey. The prevalence of AP in endodontically treated teeth in the present study was 53.1% concurring with the results of other cross-sectional studies in Scotland (51%),^14^ Canada (51%),^15^ Denmark (52%)^8^ and Turkey (53%).^9^ This prevalence was lower than those reported in Spain (65.8%)^13^ and Germany (61%).^16^ Our results demonstrated that the number of teeth with AP were 226, representing ~1.9% of the total. The frequency of teeth with AP in other surveys varied from 0.6 to 9.8%. The discrepancies observed between the results of different studies might be explained by the following aspects: (i) Lack of homogeneity of the populations being compared, (ii) lack of standardization of the methods of radiographic assessment, (iii) use of teeth or individuals as referential, (iv) quality of endodontic treatment rated by either general dentists or endodontists and (v) different levels of endodontic practice and infection control in the different populations. Some aspects relative to AP should be taken into consideration. By definition, AP consists of the inflammation of the periodontal tissues at the root apex and presents distinct pathological stages of development. Nair et al,^17^ analyzed 256 human periapical lesions and diagnosed 35% of them as periapical abscess, 50% as granuloma and 15% as cysts (9% apical true cysts, 6% apical pocket cysts).

Sjogren et al^18^ evaluated the factors that would affect the long-term outcomes of root canal therapy 8 to 10 years after the treatment. The success rate for cases with vital or nonvital pulps, but having no periapical radiolucency, exceeded 96%, whereas 86% of the cases with pulp necrosis and periapical radiolucency showed apical healing.

In this study, OPG radiographs were used for evaluating the quality of endodontically treated teeth. The radiographic measures of ‘length and density of root filling’ can be used as indicators to assess RCTs capacity to prevent recontamination and it may substitute clinical measures that assess the quality of RCT. Unfortunately, the criteria for judging the quality of RCT have not been well defined. Acceptable RCT was defined as having ‘adequate length and density of root filling’. These subjective assessments have not been standardized or calibrated; however, the results of these subjective assessments showed that ‘acceptable RCT’ had significantly lower AP than those judged ‘unacceptable’.^19^ Furthermore, it has been contended that periapical diagnosis from OPGs may result in underestimation of the real prevalence of AP as there are certain limitations of the radiographic assessment as a study method. One of these limitations involves the evaluation of the quality of root canal filling and CR based on a two-dimensional image of three-dimensional structures. Radiographic images have been used to indicate the presence of periapical infection or coronal leakage, consisting of an important diagnostic resource. Previous studies have also employed periapical radiographs with the same purpose for this study. However, research has indicated good association between OPGs and intraoral radiographs, and even a slight overestimation.^20-22^ It is therefore probable that the validity of recording AP based on OPGs is satisfactory.

Individuals with 10 or fewer remaining teeth were not included in this survey as they often had poor oral health and periodontal diseases and it was difficult to determine the influence of RCT on the incidence of radiographic AP. It was also found that there were more males undergoing endodontic treatment as compared to the females. However, gender had no significant effect on the presence of AP.^23^The total number of endodontically treated teeth was ~3% which is similar to other surveys.^8,13,15,21,23^

Recent epidemiological surveys have further investigated the significance of CR and suggested that the quality of CR may affect the outcome of RCT.^24^ Our results demonstrated that AP was present in approximately 37% of the teeth which had an adequate CR compared to 83% of the teeth which did not have an adequate CR. Based on this finding, provision of CR should be considered as the final step of the RCT to prevent postoperative recontamination.

Also, the combination of adequate CR and adequate root filling resulted in significantly reduced incidence of AP (21.6%) compared to the presence of AP (97%) when both parameters scored as inadequate. AP was four times more likely to be present in unacceptable RCTs, and three times more likely in inappropriate CR compared with the appropriate ones. The odds of finding AP in unacceptable RCT/inappropriate CR cases was >5 times greater than acceptable RCT/appropriate CR.

## CONCLUSION

The results demonstrated that a well-performed RCT and well-sealing CR are both essential for the overall success of endodontic treatment. Therefore, a considerable improvement in the quality of RCT and CR in general dental practice is required to promote oral/periapical health.

## References

[1] Hargreaves KM., Cohen S, Berman LH (2010). Cohen's pathways of the pulp..

[2] Ingle JI (2008). Ingle's endodontics..

[3] Jimenez-Rubio A, Segura JJ, Feito JJ (1998). A case of combined dental development abnormalities: Importance of a thorough examination.. Endod Dent Traumatol.

[4] Friedman S, Mor C (2004). The success of endodontic therapy-healing and functionality.. J Calif Dent Assoc.

[5] Chen SC, Chueh LH, Hsiao CK, Tsai MY, Ho SC, Chiang CP (2007). An epidemiologic study of tooth retention after nonsurgical endodontic treatment in a large population in Taiwan.. J Endod.

[6] Eriksen HM, Kirkevang LL, Petersson K (2002). Endodontic epidemiology and treatment outcome: General considerations.. Endod Topics.

[7] Imfeld TN (1991). Prevalence and quality of endodontic treatment in an elderly urban population of Switzerland.. J Endod.

[8] Kirkevang LL, Hörsted-Bindslev P, ørstavik D, Wenzel A (2001). Frequency and distribution of endodontically treated teeth and apical periodontitis in an urban Danish population.. Int Endod J.

[9] Sunay H, Tanalp J, Dikbas I, Bayirli G (2007). Cross-sectional evaluation of the periapical status and quality of root canal treatment in a selected population of urban Turkish adults.. Int Endod J.

[10] Tronstad L, Asbjornsen K, Doving L, Pedersen I, Eriksen HM (2000). Influence of coronal restorations on the periapical health of endodontically treated teeth.. Endod Dent Traumatol.

[11] Hommez GM, Coppens CR, De Moor RJ (2002). Periapical health related to the quality of coronal restorations and root fillings.. Int Endod J.

[12] Petersson K, Petersson A, Olsson B, Hakansson J, Wennberg A (1986). Technical quality of root fillings in an adult Swedish population.. Endod Dent Traumatol.

[13] Eriksen HM, Bjertness E, Orstavik D (1988). Prevalence and quality of endodontic treatment in an urban adult population in Norway.. Endod Dent Traumatol.

[14] Saunders WP, Saunders EM (1998). Prevalence of periradicular periodontitis associated with crowned teeth in an adult Scottish subpopulation.. Br Dent J.

[15] Dugas NN, Lawrence HP, Teplitsky PE, Pharoah MJ, Friedman S (2003). Periapical health and treatment quality assessment of root- filled teeth in two Canadian populations.. Int Endod.

[16] Weiger R, Hitzler S, Hermle G, Löst C (1997). Periapical status, quality of root canal fillings and estimated endodontic treatment needs in an urban German population.. Endod Dent Traumatol.

[17] Ramachandran Nair PN, Pajarola G, Schroeder HE (1996). Types and incidence of human periapical lesions obtained with extracted teeth.. Oral Surg Oral Med Oral Pathol Oral Radiol Endod..

[18] Sjogren U, Hagglund B, Sundqvist G, Wing K (1990). Factors affecting the long-term results of endodontic treatment.. J Endod.

[19] Ng YL, Mann V, Rahbaran S, Lewsey J, Gulabivala K (2008). Outcome of primary root canal treatment: Systematic review of the literature - Part 2. Influence of clinical factors.. Int Endod J.

[20] Ahlqwist M, Halling A, Hollender L (1986). Rotational panoramic radiography in epidemiological studies of dental health. Comparison between panoramic radiographs and intraoral full mouth surveys.. Swed Dent J.

[21] Eriksen HM, Berset GP, Hansen BF, Bjertness E (1995). Changes in endodontic status 1973-1993 among 35-year-olds in Oslo, Norway.. Int Endod J.

[22] Muhammed AH, Manson-Hing LR, Ala B (1982). A comparison of panoramic and intraoral radiographic surveys in evaluating a dental clinic population.. Oral Surg Oral Med Oral Pathol.

[23] De Cleen MJ, Schuurs AH, Wesselink PR, Wu MK (1993). Periapical status and prevalence of endodontic treatment in an adult Dutch population.. Int Endod J.

[24] Asgary S, Shadman B, Ghalamkarpour Z, Shahravan A, Ghoddusi J, Bagherpour A, Akbarzadeh Baghban A, Hashemipour M, Ghasemian Pour M (2010). Periapical status and quality of root fillings and coronal restorations in Iranian population.. Int Endod J.

